# Cu-Cu Thermocompression Bonding with a Self-Assembled Monolayer as Oxidation Protection for 3D/2.5D System Integration

**DOI:** 10.3390/mi14071365

**Published:** 2023-06-30

**Authors:** Maria Lykova, Iuliana Panchenko, Martin Schneider-Ramelow, Tadatomo Suga, Fengwen Mu, Roy Buschbeck

**Affiliations:** 1 Fraunhofer Institute for Electronic Nanosystems ENAS, 09126 Chemnitz, Germany; 2 Institute of Electronic Packaging Technology (IAVT), TU Dresden, 01062 Dresden, Germany; 3 All Silicon System Integration Dresden (ASSID), Fraunhofer Institute for Reliability and Microintegration (IZM), 01468 Dresden, Germany; 4 Fraunhofer Institute for Reliability and Microintegration (IZM), 13355 Berlin, Germany; 5 Collaborative Research Center, Meisei University, Tokyo 191-8506, Japan; 6 Department of Precision Engineering, Graduate School of Engineering, The University of Tokyo, 7-3-1 Hongo, Bunkyo-ku, Tokyo 113-8656, Japan; 7 SABers Co., Ltd., Tianjin 300450, China; 8 Center for Microtechnologies (ZfM), Technical University of Chemnitz, 09126 Chemnitz, Germany

**Keywords:** Cu-Cu bonding, thermocompression bonding, self-assembled monolayers, SAM, SAM desorption

## Abstract

Cu-Cu direct interconnects are highly desirable for the microelectronic industry as they allow for significant reductions in the size and spacing of microcontacts. The main challenge associated with using Cu is its tendency to rapidly oxidize in air. This research paper describes a method of Cu passivation using a self-assembled monolayer (SAM) to protect the surface against oxidation. However, this approach faces two main challenges: the degradation of the SAM at room temperature in the ambient atmosphere and the monolayer desorption technique prior to Cu-Cu bonding. In this paper, the systematic investigation of these challenges and their possible solutions are presented. The methods used in this study include thermocompression (TC) bonding, X-ray photoelectron spectroscopy (XPS), shear strength testing, scanning electron microscopy (SEM), and energy dispersive X-ray spectroscopy (EDX). The results indicate nearly no Cu oxidation (4 at.%) for samples with SAM passivation in contrast to the bare Cu surface (27 at.%) after the storage at −18 °C in a conventional freezer for three weeks. Significant improvement was observed in the TC bonding with SAM after storage. The mean shear strength of the passivated samples reached 65.5 MPa without storage. The average shear strength values before and after the storage tests were 43% greater for samples with SAM than for the bare Cu surface. In conclusion, this study shows that Cu-Cu bonding technology can be improved by using SAM as an oxidation inhibitor, leading to a higher interconnect quality.

## 1. Introduction

Over the past 60 years, electrical components have become smaller in size while their functions expanded. Moore’s Law, formulated in the 1960s, asserts that the quantity of transistors on a chip should double approximately every 18 months [1]. However, in 2016, the continuation of this trend was questioned due to various factors, including limitations in lithography techniques and challenges related to heat dissipation [2]. Current research efforts are primarily directed towards diversifying electrical components or enhancing their capabilities, known as the “more than Moore” approach or heterogeneous integration [3]. Although further miniaturization of smartphones may not be a primary concern at this stage, there is indeed a growing demand for increased functionalities and reduced fabrication costs. One of the approaches gaining traction is the use of chiplets. Chiplets are individual integrated circuit blocks that possess specific functionalities and are designed to be integrated onto a larger die alongside other chiplets [4]. Each chiplet is designed and manufactured independently, focusing on a specific functionality or component. For example, one chiplet may be responsible for processing, another for memory, and another for communication. These chiplets can be fabricated using different technologies, processes, or even by different manufacturers. For example, chiplets are already used in such products as the Ponte Vecchio graphic processing unit (GPU) from Intel and the EPYC Milan-X Processor from AMD [3]. The integration of 3D and 2.5D systems provides a way to densely connect these integrated chiplets or dies using an Si interposer with Through-Si-Vias (TSVs) [3,5].

The implementation of 2.5D and 3D integration systems for electrical components has played a significant role in increasing functionalities and reducing fabrication costs in the semiconductor industry. The use of solderless interconnects, such as Cu, enables fine pitch, high mechanical strength, and electrical conductivity. However, one of the biggest challenges in using Cu as a microcontact material for die-to-die interconnects is its susceptibility to oxidation, which can cause a higher contact resistance and hinder the formation of reliable interconnects during the Cu-Cu bonding process [6,7].

There are various solutions to this problem, including etching by formic acid directly in the bonding chamber [6], wet etching [7], plasma treatments [8,9], and passivation with permanent or temporary protective layers. A complex control system for the chemistry in the bonder is required for the formic-acid approach. Wet etching is a simple technique, but it cannot avoid re-oxidation of Cu during the transportation to the bonding chamber. Plasma treatments require complex and costly equipment. Permanent coatings are usually thin metal coatings (i.e., Au [10], Pd [11], Al [12], electroless Ni—immersion Au or ENIG [13], etc.). In the case of Au and Pd, there is an issue of fast diffusion of Cu into the passivating metal, which causes Kirkendall voids and reliability issues. In the case of Al and ENIG, the resistivity of the interconnect increases due to these coatings.

Organic solderability preservative (OSP) is one of the most common temporary coatings for Cu. However, the literature lacks detailed information about OSP solutions specifically for downscaled interconnects, possibly due to concerns about contamination issues [14]. Furthermore, there remains the challenge of re-oxidation after OSP removal and the renewed adsorption of organic contaminants when Cu is exposed to air even for a few seconds due to its high reactivity. Passivation with the self-assembled monolayer (SAM) is reported to be suitable for fine-pitch interconnects and is followed by in situ thermal annealing before bonding to remove the monolayer [15,16]. Therefore, it was decided to investigate this passivation method in detail.

Self-assembled monolayers (SAMs) are densely packed monomolecular layers formed by the spontaneous adsorption of organic molecules onto a substrate surface. SAMs can be classified into two main categories: short-chain SAMs and long-chain SAMs, based on the length of the carbon backbone in the molecules forming the SAM. This technique helps to protect Cu from oxidation and enhance the reliability of interconnects in 2.5D and 3D integration systems. Normally, SAM passivation and bonding are carried out on different days at different places. Therefore, the passivated samples are exposed to several days/weeks of air exposure at room temperature. The monolayer is susceptible to degradation when exposed to air at room temperature, which has prompted investigations into methods of prolonging its function. Long-chain self-assembled monolayers (SAMs) are more effective oxidation inhibitors, but they are more difficult to remove before bonding than short-chain SAMs [17]. Storage at low temperatures is a promising technique for prolonging the function of the monolayer, although its effectiveness has not been thoroughly studied for SAMs with short backbones [18,19,20]. Furthermore, the impact of storage at low temperatures on the quality of bonding after prolonged periods remains an unresolved question.

Smooth, sputtered surfaces are commonly recommended for SAM passivation [21], but Cu microbumps are typically electroplated and have a greater roughness than those of sputtered surfaces. The quality of electrochemically deposited (ECD) Cu is of great importance for SAM passivation and subsequent bonding. The formation of well-ordered SAM structures can be influenced by various factors, including the presence of vacancy islands, monoatomic step-edges, and grain boundaries with deep crevices on the metal surface. These features can disrupt the regular arrangement of SAM molecules and hinder the formation of a uniform and ordered monolayer. Impurities on the metal surface can disrupt the adsorption process and interfere with the formation of a well-ordered SAM structure [21]. Therefore, the protective effect of SAMs on electroplated Cu is addressed in this paper.

The studies by Peng et al. and Tan et al. focus on the investigation of the thermocompression (TC) bonding of Cu with SAM passivation using pre-heating for in situ desorption [17,22]. In these studies, polished Cu pads were coated with a layer of 1-hexanethiol (C6) for protection. After the passivation process, the samples were stored in ambient air conditions for a period of three days at room temperature. Following the storage period, the passivated dies were transferred to the bonding chamber, which underwent a purging step with N_2_ gas. In the experimental setup, the samples underwent an in situ pre-heating process at a temperature of 250 °C for a duration of 10–30 min. Following the pre-heating step, the actual TC bonding process took place in a vacuum environment at a temperature of 250 °C for a duration of one hour. XPS analysis was performed to assess the cleanliness of the Cu surface after the desorption process. While the S-spectra before and after desorption were examined, it is worth noting that the provided information did not include data regarding the presence of O or C on the Cu surface after pre-heating.

Based on the literature observations [23,24], it appears that storing materials at low temperatures has the potential to stop the degradation of SAM and, hence, the oxidation of Cu. According to [18], the density of gauche conformations (defects in the SAM tails, mostly near the methyl groups) in the SAM coating decreases with temperature. Hutt et al. reported that a SAM attains a better ordering through lower temperatures because of the decrease in thermal energy, which is capable of disrupting the van der Waals forces between the hydrocarbon chains [20].

However, there are not enough experimental data available to determine the effect of low-temperature storage on Cu-Cu bonding. Different research groups have reported that SAM removal is necessary before bonding, as SAM can serve as a barrier to Cu-Cu interconnect if not removed [17]. Carbonell et al. conducted experiments to measure the desorption temperature for the desorption of 1-decanethiol (C10) through thermal desorption spectrometry (TDS) [25]. While annealing (heating rate 0.39 °C/s), they found two desorption peaks: one at 95 °C and another around 150 °C, which were interpreted as a two-step SAM desorption process. Although the maximum desorption peak was at 150 °C, its intensity decreased slowly. Hence, the full desorption of C10 took place at temperatures greater than 250 °C. Kodama et al. reported that 1-hexanethiol (C6) radicals were desorbed (heating rate 2 °C/min) at 97 °C and 187 °C [26]. No S species were found after the desorption of 1-octadecanethiol (C18) when the temperature reached 210 °C [27]. However, there are several research gaps in investigating the desorption procedure for TC bonding with SAM, such as whether the Cu surface remains free from SAM and oxide after annealing at a constant temperature, which has not been systematically addressed in the literature.

## 2. Materials and Methods

### 2.1. Sample Description

**Figure 1:**
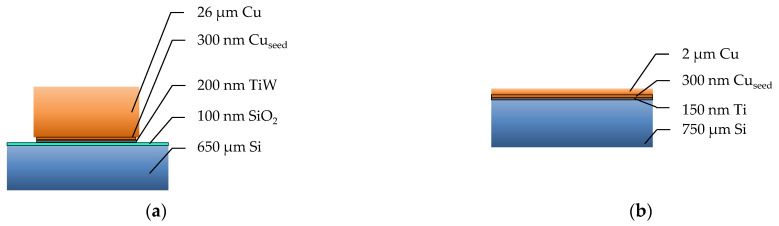
Schematic representation of the layer stacks of the top (**a**) and the bottom (**b**) dies.

The sample structure and properties are important for the comparison of bonding parameters and bonding results with other literature reports. Figure 1 shows the layer set-up for the top and bottom dies, including layer thicknesses.

Table 1 shows the main properties of the top and bottom dies.

### 2.2. Passivation Procedure

Passivation took place in a cleanroom atmosphere. The samples were first etched using a 2% dilute solution of HCl (15 mL of 37% HCl, from Sigma-Aldrich and 250 mL deionized water) for a duration of 3 to 5 min to remove the native Cu oxide. The thickness of the oxide layer is estimated to be around 2 nm [28]. The samples were kept in an N_2_-purged dry box after electroplating and were exposed to air only during transportation. Therefore, no CuO species were expected to be on the surface before etching [29]. After the wet-etching process, the samples underwent a rinse using 2-propanol (99% from Wako). Subsequently, the samples were immersed in a solution containing 1-hexanethiol (C6, from Sigma-Aldrich, Darmstadt, Germany) diluted to a concentration of 1 mMol in isopropanol. In total, 4 mL of glacial acetic acid (99.7% from Wako) was added per liter of solution to remove any Cu oxide that formed during air exposure [20,30]. Finally, the samples were rinsed with isopropanol and dried using N_2_ gas.

This study compares two types of samples: those without SAM passivation (no SAM) and those with (SAM) passivation. The uncoated dies (no SAM) are etched to remove the Cu oxide. The passivated dies (SAM) are etched and passivated with SAM.

### 2.3. Analysis of the Cu Surface, SAM Layer Quality, and Its Protective Effect

The surface roughness of the bottom dies before and after the SAM passivation was measured using atomic force microscopy (AFM, L-trace II, SII NanoTechnology Inc., Tokyo, Japan).

X-ray photoelectron spectroscopy (XPS) using JPS-9200 (Jeol) involved two steps to obtain a comprehensive understanding of the chemical state of the surface of the samples. The first step involved X-rays irradiating the Cu surface to determine its chemical state, which is represented as the “initial state”. However, Cu is always covered by an adventitious carbon contamination layer that hampers characterization of the “real” chemical composition of the Cu surface. To overcome this limitation and obtain a more accurate picture of the surface composition, a second step was performed, known as the “cleaning step”. In this step, the sample surface was subjected to low-energy (500 eV) Ar^+^ ion bombardment for a duration of 2 min, which effectively removed the organic contamination layer. Following the cleaning step, the sample surface was re-examined using XPS.2.4. 

The applied thermocompression bonding technique can be described as follows. Bonding was carried out in the N_2_-purged bonding chamber of the Alpha Design TC bonder. Table 2 shows the main bonding parameters used in these experiments. Initially, the top die was placed face-down in the center of the face-up bottom die lying on a heating stage in the bonding chamber. Then, the chamber was evacuated until the pressure reached 0.09 MPa. Subsequently, the chamber was continuously purged with Ar and retained a pressure of 0.1 MPa.

The thickness of the dies and other parameters were entered manually into the machine’s software. The stage was heated for a duration of 30 min to initiate the monolayer desorption. During this time, the chips were stacked on top of each other without any external bonding force applied. After the pre-heating step, the bonding tool started pressing the chip stack at 250 °C. The stage heating rate was set to 2 °C/s.

### 2.4. Analysis of the Interconnect Bonding Quality

Shear strength testing is a common method used to determine the strength of a bond. In this study, the PTR-1100 shear tester from RHESCA Co., Ltd. (Tokyo, Japan) was used to measure the shear strength of the Cu-Cu bonds. This shear tester had a maximum load of 50 kgf and was operated at a speed of 20 µm/s. To hold the dies in place during testing, a special clamping tool with 500-µm-high holders was used. The shear strength was calculated as the maximum force required to break the bond divided by the bond area.

Fracture surface analysis is an important step in characterizing die-to-die bonding since it provides valuable information about the bonding quality; shear strength results often have high deviations.

The SEM images and EDX analysis of the fracture surface types were performed using GAIA3 (TESCAN) equipment. The detector X-Max 150 and the analysis software Aztec 4.2 (Oxford Instruments) were used for EDX analysis.

## 3. Results

### 3.1. Influence of the SAM Passivation on the Cu Surface Roughness

**Figure 2:**
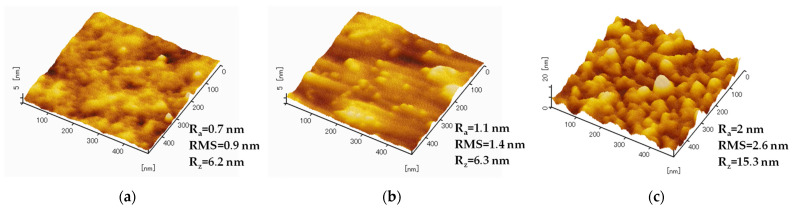
AFM images and measured values of average (R_a_), root mean square (RMS), and maximum (R_z_) roughness values of Cu surface in its initial state (**a**), after etching (**b**), and after passivation with SAM (**c**).

In order to investigate the influence of Cu surface pre-treatments on its roughness, AFM measurements were performed on the bottom dies. Figure 2 presents the results of AFM measurements for the Cu surface in its initial state (Figure 2a), after its etching by HCl 2% (Figure 2b), and after SAM passivation (Figure 2c).

The initial average Cu roughness is low (R_a_ = 0.7 nm) due to the CMP preparation step of the bottom dies. This value increases after etching with dilute HCl by 0.4 nm and elevates again by 0.9 nm after C6 passivation.

The higher the surface roughness, the more defects can be found in the SAM layer (i.e., gauche conformations). Hence, the space with ordered SAM molecules stays relatively clean (protected), whereas the unprotected spots (with defects in SAM) adsorb organic contamination from air, which can lead to a slightly increased overall roughness of the surface after SAM passivation.

Ghosh at el. reported R_a_ = 2 nm for a freshly sputtered PVD Cu surface and R_a_ = 2.5 nm for the Cu surface passivated by C3-SAM [31]. A Master’s thesis [14] showed that PVD Cu has an initial roughness of 4.4 nm after storage in cleanroom air conditions; the value increases by 0.6 nm after etching by MS6020 solution and increases again by 0.9 nm after C18 passivation.

Another Ph.D. study confirmed the elevation of the roughness after the exposure of the polished Cu surface (R_a_ = 0.5 nm) to a regular acid clean (R_a_ = 1.2 nm) [32].

The results in the present paper correlate well with the literature and show that the average roughness remains in a low range even after the passivation procedure.

### 3.2. Impact of the Desorption and Storage Parameters on the Chemical Composition of the Cu Surface

#### 3.2.1. Impact of the Desorption Parameters on the Chemical Composition of the Cu Surface

**Figure 3:**
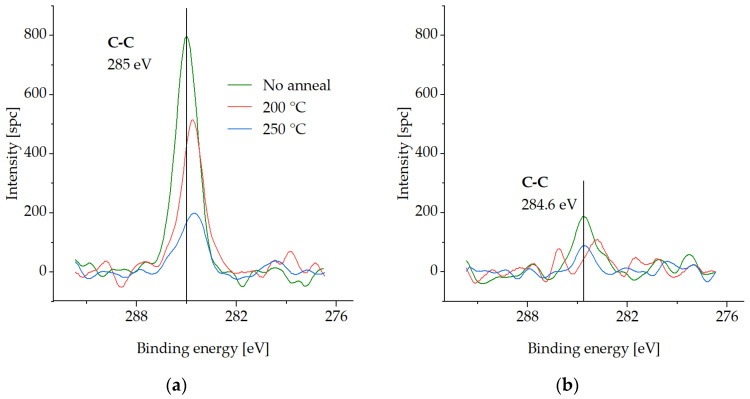
XPS spectra of C1s on the Cu surface before SAM desorption (no anneal) and after SAM desorption at 200 °C (200 °C) and at 250 °C (250 °C) for 30 min: (**a**) in the initial state, (**b**) after the cleaning step.

**Figure 4:**
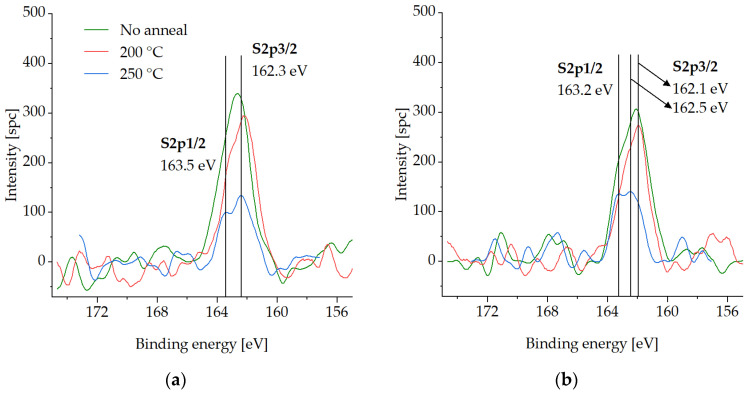
XPS spectra of S2p on the Cu surface before SAM desorption (no anneal) and after SAM desorption at 200 °C (200 °C) and at 250 °C (250 °C) for 30 min: (**a**) in the initial state, (**b**) after the cleaning step.

**Figure 5:**
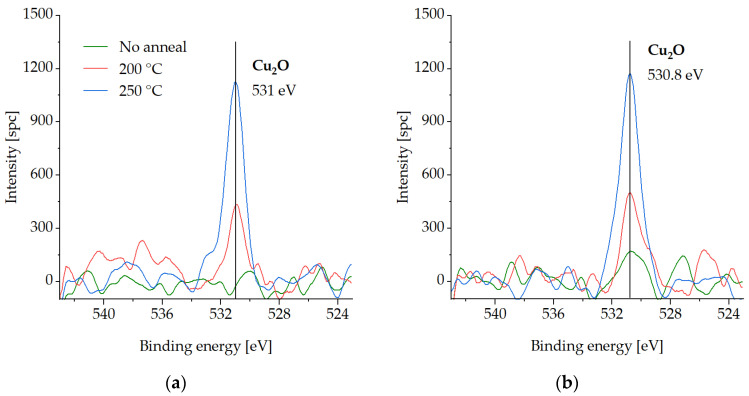
XPS spectra of O1s on the Cu surface before SAM desorption (no anneal) and after SAM desorption at 200 °C (200 °C) and at 250 °C (250 °C) for 30 min: (**a**) in the initial state, (**b**) after the cleaning step.

**Figure 6:**
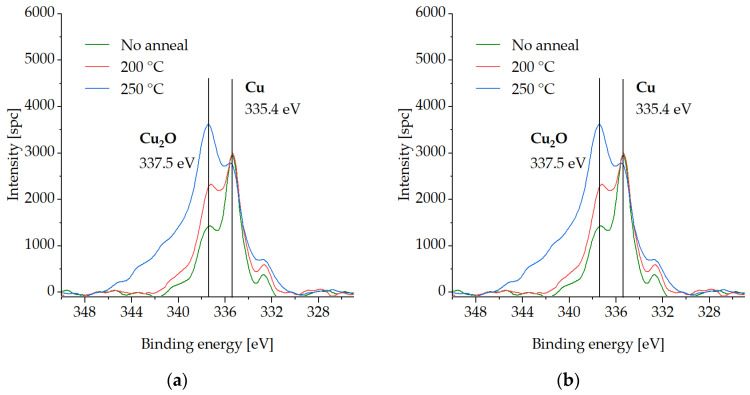
XPS spectra of Cu LMM on the Cu surface before SAM desorption (no anneal) and after SAM desorption at 200 °C (200 °C) and at 250 °C (250 °C) for 30 min: (**a**) in the initial state, (**b**) after the cleaning step.

**Figure 7:**
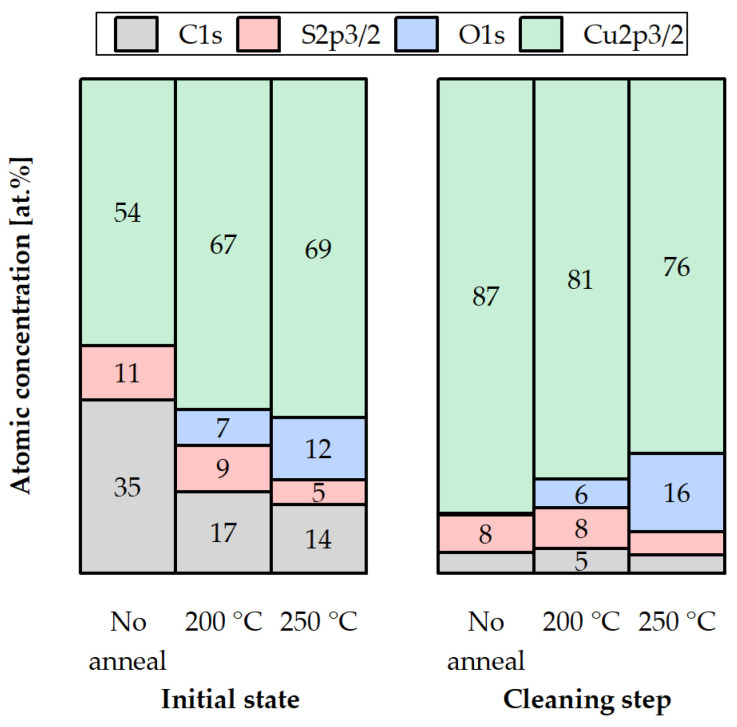
Atomic concentrations of C1s, S2p3/2, O1s, and Cu2p3/2 on the Cu surface before (no anneal) and after desorption at 200 °C (200 °C) and 250 °C (250 °C) for 30 min: in the initial state and after the cleaning step.

In this section, XPS spectra are presented for various compounds on the Cu surface that is coated with the SAM layer, following three different desorption tests in an Ar gas ambient environment. The measurements were carried out on the test samples pre-annealed at either 200 °C or 250 °C for 30 min to investigate the binding force of the SAMs to the Cu surface and their susceptibility to desorption. Samples with SAM (no desorption) were also analyzed for comparison. The samples were exposed to an atmospheric ambient environment for a maximum of two days during storage in a vacuum desiccator. XPS analysis was performed in two steps, as described in Section 2.3. To reference the spectra, the C-C peak was set at 285 eV. Figure 3, Figure 4, Figure 5 and Figure 6 show XPS spectra of C1s, O1s, S2p, and Cu LMM with and without the desorption step at 200 °C and 250 °C for 30 min. The results are summarized in Figure 7, which displays the distribution of the atomic concentrations of these elements on the Cu surface.

Figure 3 shows that the C1s intensity decreases with increasing desorption temperature. This can be explained by the gradual SAM desorption. At 287–289.1 eV, no C-O binding was detected [33].

Figure 4 highlights that the S2p spectra intensities decrease with increasing desorption temperature, which is also connected to the SAM desorption [21,34]. No oxidized S species, which would occur at >166 eV [35,36], can be detected either before or after the desorption procedures.

No O peak is detected for the sample before the SAM desorption (Figure 5). The intensity of oxygen significantly increases with temperature. The peaks at 530.8–531 eV can be attributed to the presence of Cu_2_O [35,37].

Figure 6 shows the increase in Cu_2_O at 337.2–337.5 eV with desorption temperature. Cu_2_O is normally situated at the distance of 2 eV from the Cu peak at 335.4 eV. CuO, which should lie 1-eV higher than the Cu peak, is not detected [33,38,39].

Figure 7 summarizes the atomic concentrations of the elements above before and after SAM desorption, in the initial state and after the cleaning step.

In the initial state, C1s and S2p3/2 decrease with the increasing desorption temperature. Cu2p3/2 shows a slight elevation due to the falling C1s and S2p3/2 concentrations. After the cleaning step, C1s is reduced to a level of ≤5 at.%. S2p3/2 shows 8 at.% for the samples before and after the desorption at 200 °C. Less than 5 at.% of S2p3/2 is detected after the 250 °C desorption. The Cu2p3/2 percentage decreases due to the growth of the O1s content. Either before or after the cleaning step, no oxide is detected in the sample without the pre-anneal (SAM desorption). O1s increases with temperature, which can be the result of a non-perfect Ar gas ambient environment in the bonding chamber (presence of O_2_).

No Cu oxidation was detected for the sample without anneal, which confirms that SAM passivation technique in air was performed successfully. No C or S oxidized species were found on the Cu surface after the desorption procedures, but Cu_2_O components were found, which suggests that the SAM layer does not oxidize. The C and S amounts decrease with increasing desorption temperature, which confirms partial SAM desorption. S cannot be fully removed even after annealing at the highest temperature (250 °C). The amount of Cu oxide on the Cu surface increases with increasing desorption temperature, because the inert gas atmosphere is not completely free of O. When choosing between the annealing options, 200 °C seems to be more suitable to avoid overly strong oxidation while partially removing the SAM.

#### 3.2.2. Impact of the Storage Parameters on the Chemical Composition of the Cu Surface

**Figure 8:**
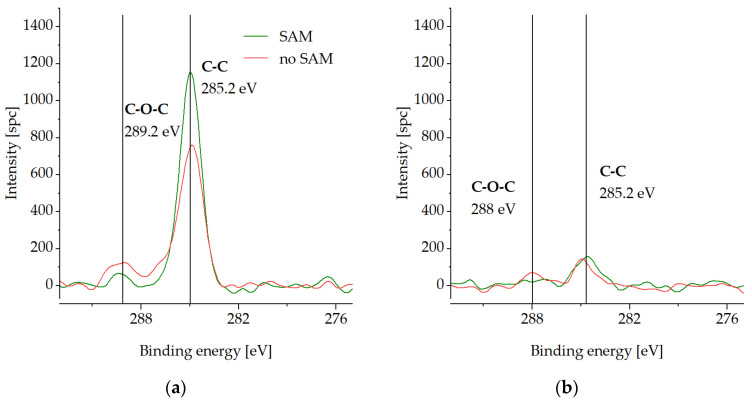
XPS spectra of C1s on the Cu surface with (SAM) and without SAM (no SAM) passivation after storage at −18 °C for 3 weeks in air: (**a**) in the initial state, (**b**) after the cleaning step.

**Figure 9:**
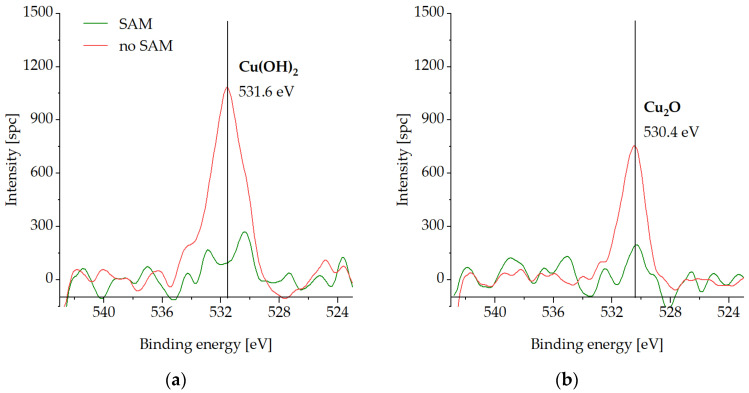
XPS spectra of O1s on the Cu surface with (SAM) and without SAM (no SAM) passivation after storage at −18 °C in a conventional freezer for three weeks: (**a**) in the initial state, (**b**) after the cleaning step.

**Figure 10:**
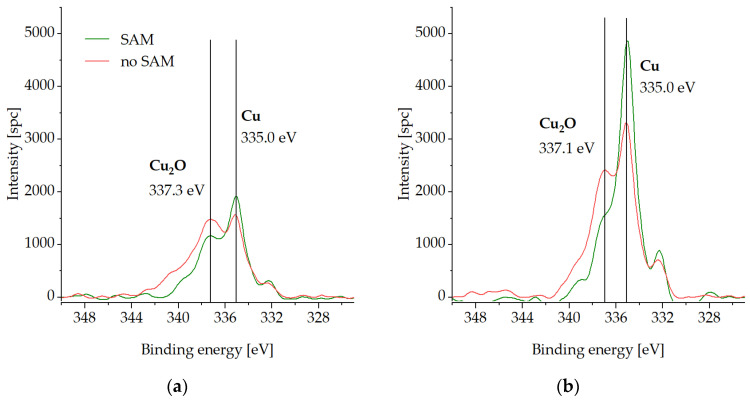
XPS spectra of Cu LMM on the Cu surface with (SAM) and without SAM (no SAM) passivation after storage at −18 °C in a conventional freezer for three weeks: (**a**) in the initial state, (**b**) after cleaning step.

**Figure 11:**
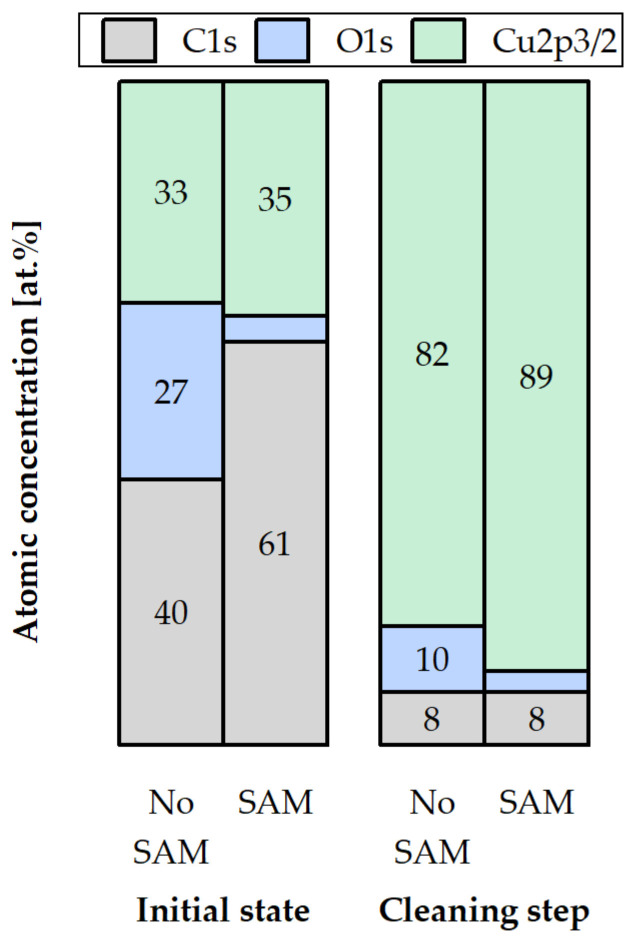
Atomic concentrations of C1s, S2p3/2, O1s, and Cu2p3/2 on the Cu surface with (SAM) and without SAM (no SAM) passivation after storage at −18 °C in a conventional freezer for three weeks: in the initial state (initial state) and after the low-energy Ar^+^ ion bombardment step (cleaning step).

The bottom dies with and without the SAM passivation were stored at −18 °C in a conventional freezer for three weeks. Figure 8, Figure 9, Figure 10 and Figure 11 present the XPS analysis of the chemical state of the Cu surface after storage.

In the initial state (Figure 8a), the intensity of C1s is higher for the SAM sample because of the C-backbone of the monolayer. Both samples exhibit a slight peak at 289.2 eV which corresponds to O-C=O bindings [40], although the intensity of the peak is lower for the SAM sample. It is worth noting that the SAM samples without storage, as well as the annealed samples (Figure 3), do not show any of the O-C=O bindings. This is typical only for the samples exposed to the atmosphere at a low temperature for a longer time.

The C species are almost completely removed after the cleaning step (Figure 8b). The uncoated sample exhibited a shifted slight peak at 288 eV, which corresponds to C=O bindings [33].

Figure 9 presents the O1s spectra on the Cu surface. Cu(OH)_2_ is detected for the unprotected sample in the initial state (Figure 9a). This can be explained by the adsorption of OH-bindings from the air moisture during the sample transportation and storage. The cleaning step reveals the shift to the Cu_2_O peak at 530.4 eV for the unprotected sample (Figure 9b). Almost no oxygen content is observed for the passivated die before or after the cleaning step, which confirms the excellent SAM protective function during the storage conditions described above.

Cu LMM spectra are highlighted on Figure 10. The sample without the SAM passivation exhibits elevated peaks at 337.3 eV (Cu_2_O), in contrast to the samples with SAM passivation for both the initial state and the cleaning step.

Figure 11 summarizes the distribution of the atomic concentrations of the elements above on the Cu surface after the storage test. In the initial state, the C-percentage is higher for the SAM sample (61 at.%) in comparison to the uncoated sample (40 at.%), which is caused by the CH-backbone of the thiolate. The O concentration is 27 at.% for the die without SAM in comparison to 4 at.% for the passivated die. After the cleaning step, 10 at.% of the O species is left on the Cu surface for the sample without SAM, whereas the coated sample exhibits 3 at.%. The amount of C is 8 at.% for both dies after the cleaning step.

When comparing these results to the desorption analysis in the initial state (Figure 7), it is noticeable that the oxygen content on the stored sample without SAM is much higher (27 at.%) in comparison to the SAM samples, annealed at 200 °C (7 at.%) and 250 °C (12 at.%) in the non-ideal inert gas atmosphere. Observing the cleaning step in Figure 7 and Figure 11, the stored SAM sample exhibits barely 3 at.% of O, almost the same as the oxide-free SAM sample without storage or annealing. The O content of the stored sample without SAM (10 at.%) is approximately 1.7-times higher than the annealed SAM sample at 200 °C (6 at.%) and nearly 1.5-times lower than the annealed SAM sample at 250 °C (16 at.%). Therefore, the choice of 200 °C as an annealing temperature for desorption seems to be the most appropriate under the given conditions.

Tan et al. report on the O content at the bonding interface after three days of storage at room temperature in an atmospheric ambient environment for both sample types, with and without SAM. For the uncoated Cu, O diffuses into Cu and seems to have a higher atomic concentration than the SAM interface [22]. For the SAM-coated wafers, O is also present at the bonding interface and does not diffuse into Cu. No evaluation of the S content has been conducted at the bonding interface or before bonding.

The XPS results of Peng Lan’s dissertation confirm that the O content is much lower for SAM/Cu in comparison to the unprotected Cu after 12 days of storage at room temperature [32].

### 3.3. Impact of the Desorption and Storage Parameters on the Shear Strength of the Cu-Cu Interconnects

#### 3.3.1. Proposed Classification of the Fracture Surfaces after the Shear Strength Tests

**Figure 12:**
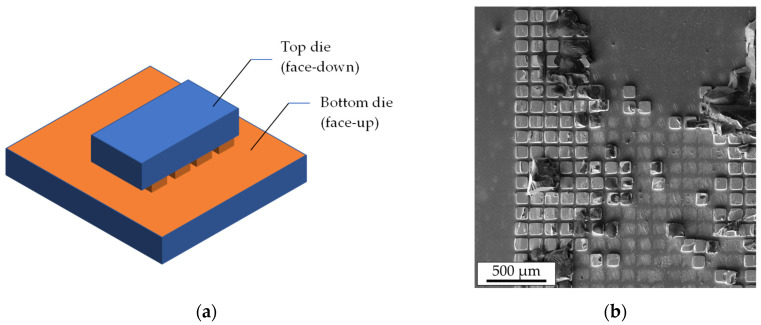
The 3D schematics of the top and bottom dies after TC bonding (**a**); SEM image of a part of a bottom die surface after the shear strength test (**b**).

Shear strength characterization comprises fracture surface distribution on a die because it delivers more precise information about the mechanical strength and the weakest areas of the interconnects. Figure 12a shows a 3D schematic view of the bonded dies, while Figure 12b presents a part of a bottom die after the shear strength test. Certain microbumps stuck to the bottom die, while some of them remained on the top die. This suggests a varying distribution of the fracture surfaces throughout the interconnects of a single die.

**Figure 13:**
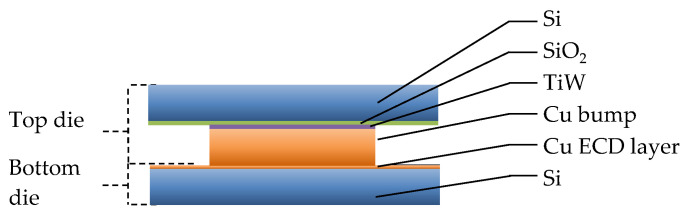
Schematic simplified representation of the layer stack of a single interconnect of the bonded top and bottom dies before the shear strength test.

Fracture surface analysis is carried out using SEM and EDX. Figure 13 shows the simplified schematic layer stack of a bonded interconnect in the cross-section. 

**Figure 14:**
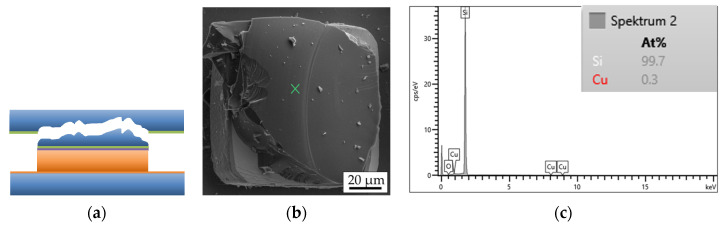
Si fracture surface type of a single interconnect: (**a**) schematic view, (**b**) SEM image of the bottom side, (**c**) EDX graph of the marked position on the SEM image.

**Figure 15:**
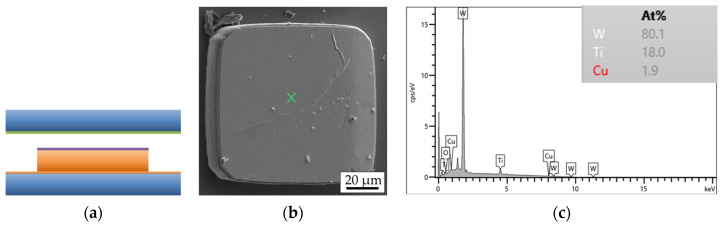
TiW fracture surface type of a single interconnect: (**a**) schematic view, (**b**) SEM image of the bottom side, (**c**) EDX graph of the marked position on the SEM image.

**Figure 16:**
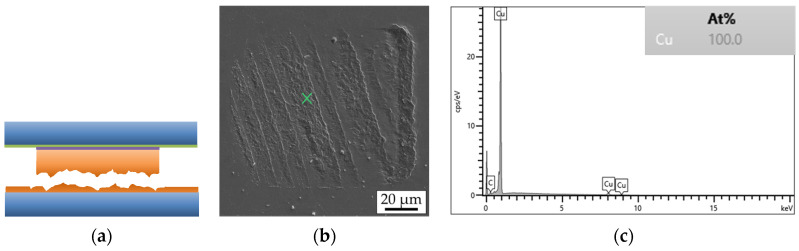
Cu fracture surface type of a single interconnect: (**a**) schematic view, (**b**) SEM image of the bottom side, (**c**) EDX graph of the marked position on the SEM image.

**Figure 17:**
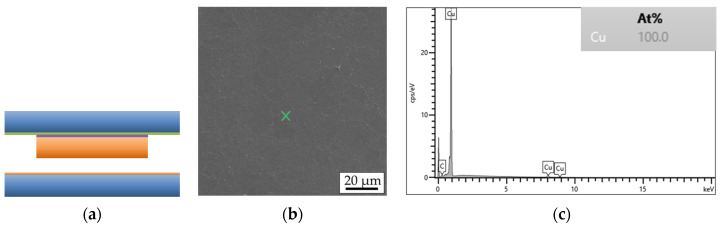
No-contact fracture surface type of a single interconnect: (**a**) schematic view, (**b**) SEM image of the bottom side, (**c**) EDX graph of the marked position on the SEM image.

Fracture through Si (Figure 14a) or an adhesive layer of TiW (Figure 15a) suggests a strong interconnect, indicating a higher Cu-Cu shear strength. These types of fracture are referred to as “Si” or “TiW” fractures, respectively. On the other hand, fracture through the Cu-Cu bonding interface (Figure 16a) suggests a reduced mechanical strength of the interconnect. When the microbumps are not contacting the bottom die, no interconnect is formed, and this type of fracture surface is called “no contact” (Figure 17a). The “no contact” fracture type most likely exists because of the non-planarity between the bonding tool and the stage.

The “no contact” area is detected in the upper right die corner for most of the bonded sample pairs (the orientation of the dies was marked). This suggests that there may be a slight non-planarity between the bonding stage and head, which is a known issue for bonding in the literature [41,42,43]. This could lead to variations in fracture surface types and bonding strength across the sample pairs.

The calculation of shear strength in MPa involves dividing the maximum shear force in N by the initial bonding area. The “no contact” area is not taken into account during the estimation of shear strength in MPa.

#### 3.3.2. Impact of the Desorption Parameters on the Shear Strength of the Cu-Cu Interconnects

**Figure 18:**
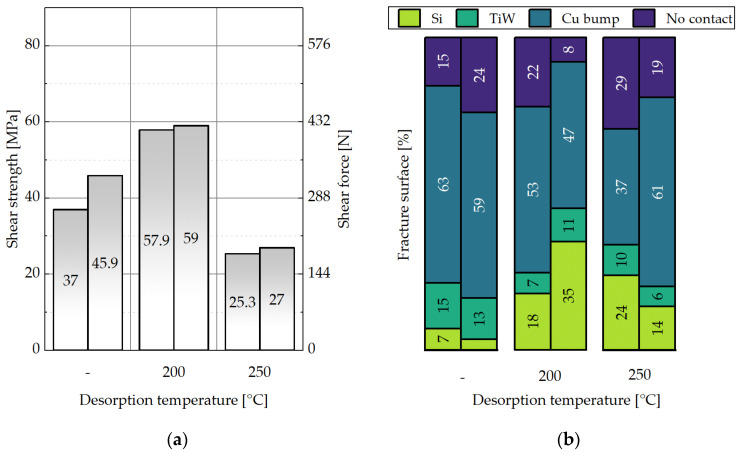
Representation of no SAM desorption (-) in comparison to SAM desorption at 200 °C and 250 °C for 30 min. Influence of these parameters on: (**a**) the shear strength, (**b**) the distribution of the fracture surface types.

The shear strength tests are performed on the interconnects after the bonding procedure to characterize their mechanical strength. Figure 18 shows the results of these tests and the distribution of the fracture surfaces on the bottom dies. Each column represents one sample pair. The data indicate that oxide growth, which increases with higher desorption temperatures, affects the shear strength of the interconnects. The samples with a 250 °C desorption exhibit the lowest shear strength values in the range of 25–27 MPa, while those with a 200 °C desorption temperature have the highest values in the range of 58–59 MPa. Interestingly, samples with SAM (no desorption) can also be bonded with relatively high shear strength values of 37–46 MPa; however, these samples have a lower percentage of Si fractures of 5–7% compared to the other samples with values of 14–35%. The highest rate of Si and TiW fractures is observed for the samples with desorption at 200 °C.

Lim et al. reported that Cu-Cu wafer bonding without SAM desorption (no pre-anneal at 250 °C) leads to a poor bonding quality [44,45]. The results in the present paper for the samples with SAM (no desorption) are better than the results after desorption at 250 °C. Firstly, for the die-to-die (D2D) bonding, the SAM desorption is probably not as significant as for the wafer-to-wafer (W2W) bonding. D2D usually requires a higher bonding pressure and, therefore, Cu atoms can break through the SAM monolayer for the Cu-Cu interdiffusion. Additionally, it is easier for the monolayer to be “squeezed out” through the 6.2-mm die edges in contrast to the 150-mm wafers. The C-SAM images of the wafers show that the wafer edges (≥10 mm) are bonded well, which correlates with the explanation above. Secondly, the absence of the pre-anneal in an imperfect Ar gas atmosphere may be beneficial for the shear strength of SAM/Cu without desorption, as no Cu oxidation takes place. Nevertheless, the presence of SAM at the bonding interface can impede the interconnect quality after the aging and reliability tests.

A desorption temperature of 250 °C is also used in other studies [17,22,46,47]. A certain amount of O content is still present at the bonding interface even after desorption in one of these reports [22]. In the present paper, it is confirmed that a lower temperature (200 °C) can deliver a higher bond strength due to less re-oxidation of Cu in the bonding chamber and the partial removal of SAM. This is beneficial for the quality of the Cu-Cu interconnects and, at the same time, for the temperature-sensitive components during bonding. 

#### 3.3.3. Impact of the Storage Parameters on the Shear Strength of the Cu-Cu Interconnects

**Figure 19:**
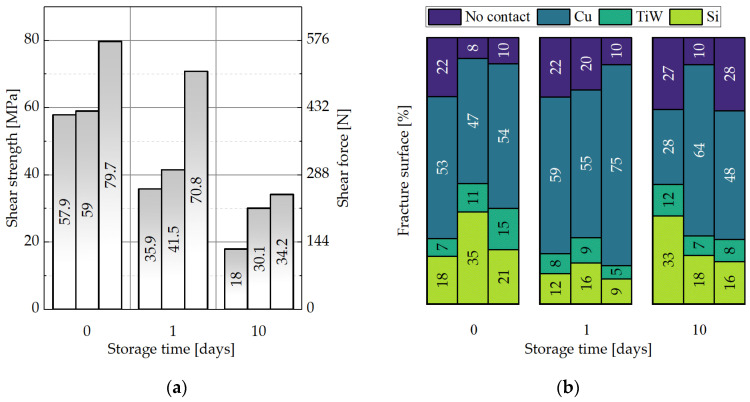
Influence of the storage time for 0, 1, and 10 days at −18 °C for the dies with the SAM coating on: (**a**) the shear strength, (**b**) the distribution of the fracture surface types.

**Figure 20:**
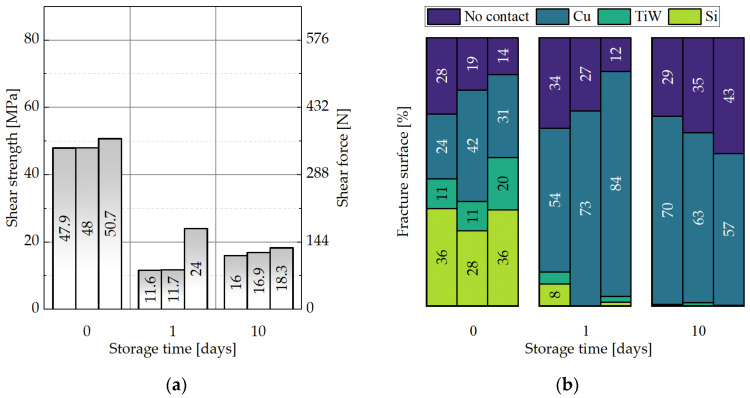
Influence of the storage time for 0, 1, and 10 days at −18 °C for the dies without the SAM coating on: (**a**) the shear strength, (**b**) the distribution of the fracture surface types.

Figure 19a and Figure 20a present that prior to storage, the samples with SAM show values of 58–80 MPa and the dies without SAM deliver 48–51 MPa of shear strength. Here also, each column represents one sample pair. After one day of storage at −18 °C in an ambient atmosphere, the values without SAM significantly drop down to 12–24 MPa, while the SAM dies show values of 36–71 MPa. After 10 days of storage, the coated dies still present values of 18–34 MPa, while the uncovered samples provide only 16–18 MPa. The obvious trend is towards a decrease in the shear strength with the storage duration for both sample types. However, after 10 days of storage, the average die shear strength is 38% greater for the Cu/SAM sample than for the sample without the SAM coating. Overall, the average shear strength for all the SAM samples in the storage experiment is 43% higher than the mean shear strength of the bare Cu.

No significant differences could be observed for the samples with SAM regarding the distribution of fracture surfaces (Figure 19b). For the bare Cu samples, the amount of Si and TiW fractures almost disappeared after one and ten days of storage in the freezer (Figure 20b). This indicates that the oxidation of Cu during storage can lead to a reduced interconnect quality in the absence of a protective SAM layer.

Tan et al. reports a shear strength of 54–66 MPa for the chips (bonding of pads to blanket Cu) with SAM and 9–11 MPa for the uncoated Cu after three days of storage in a cleanroom atmosphere [22]. The desorption temperature was kept at 250 °C for 10 min in an N_2_ inert gas atmosphere. After that, the wafers were bonded at 250 °C at 250 kPa for one hour. The authors evaluated only those samples with Cu-Cu fractures. The variation in the shear strength was reported to be caused by the bonding non-uniformity.

Peng reported on two types of fracture surfaces in his thesis: TaN-Cu and Cu-Cu [32]. The C6-SAM desorption was conducted at 250 °C in an N_2_-purged chamber, followed by wafer bonding at 350 °C for one hour, and annealing at 350 °C for a further hour in a vacuum. The author also suggests that the failure between the TaN and the Cu layers points out strong Cu-Cu interconnects.

## 4. Conclusions

Overall, the study demonstrates the effectiveness of using short-chain SAM (C6) passivation on Cu surfaces to improve the quality of Cu-Cu bonding technology. The shear strength of the bonded dies with SAM is significantly higher than that of the freshly etched dies without the SAM coating. Precise O control in the bonding chamber might be of importance during the desorption procedure, as the residual O content can cause re-oxidation of the SAM-free Cu surface. In our paper, Cu shows slight oxidation after the desorption procedure, along with partial SAM desorption. Cu oxidation, as well as SAM desorption, tends to increase with temperature. The Cu/SAM surface contains almost no O after three weeks of storage at −18 °C in an ambient atmosphere, in contrast to the unprotected samples. The degradation of the shear strength with storage time is significantly retarded by SAM passivation. The presence of Si and TiW fractures and the lower percentage of Cu fractures in the interconnects with SAM coating suggest a lower oxidation of the Cu surface during the storage time, which improves the interconnect quality and the Cu-Cu bonding technique in general. 

## Figures and Tables

**Table 1:** Characteristic parameters of the top dies.

Parameter	Top Die	Bottom Die
Die size	3.35 × 6.2 mm^2^	10 × 10 mm^2^
Bump height/Cu thickness	26 µm	2 µm
Cu type	ECD	ECD
Bump size or bump diameter	100 × 100 µm^2^	-
Bump form	square	-
Bump pitch	130 µm	-
Bump quantity	720	-
Bump matrix	18 × 40	-
Bonding area	7.2 mm^2^	-
Planarization	fly cut	CMP
Roughness	R_a_ = 60 nm, R_z_ = 439 nm	R_a_ = 0.7 nm, R_z_ = 6 nm
Cu texture	{001}	{001}, {111}

**Table 2:** Main bonding parameters.

Bonding Parameter	TC Bonding
Set bonding pressure	40 MPa
Set bonding temperature	250 °C
Bonding time	30 min
Bonding area	7.2 mm^2^
Desorption temperature	200 °C; 250 °C
Desorption time	0 min; 30 min

## Data Availability

The data presented in this study are available on request from the corresponding author.

## References

[1] Tummala R.R. (2001). Fundamentals of Microsystems Packaging.

[2] Waldrop M.M. (2016). More Than Moore. Nature.

[3] Zhang S. (2022). Challenges and recent prospectives of 3D heterogeneous integration. e-Prime–Adv. Electr. Eng. Electron. Energy.

[4] Veres J., Bringans R.D., Chow E.M., Lu J.P., Mei P., Ready S.E., Schwartz D.E., Street R.A. (2017). Additive manufacturing for electronics “Beyond Moore”. Technical Digest–International Electron Devices Meeting, IEDM.

[5] Kim J., Murali G., Park H., Qin E., Kwon H., Chaitanya V., Chekuri K., Dasari N., Singh A., Lee M. (2019). Architecture, chip, and package co-design flow for 2.5D IC design enabling heterogeneous IP reuse. Proceedings of the Design Automation Conference.

[6] He R., Fujino M., Akaike M., Sakai T., Sakuyama S., Suga T. (2017). Combined surface activated bonding using H-containing HCOOH vapor treatment for Cu/Adhesive hybrid bonding at below 200 °C. Appl. Surf. Sci..

[7] Panigrahy A.K., Chen K.N. (2018). Low Temperature Cu-Cu Bonding Technology in Three-Dimensional Integration: An Extensive Review. J. Electron. Packag. Trans. ASME.

[8] Yamamoto M., Higurashi E., Suga T., Sawada R., Itoh T. (2018). Properties of various plasma surface treatments for low-temperature Au–Au bonding. Jpn. J. Appl. Phys..

[9] Wang C., Suga T. A novel room-temperature wafer direct bonding method by fluorine containing plasma activation. Proceedings of the 2010 60th Electronic Components and Technology Conference (ECTC).

[10] Lei H., Tang Y.-J. (2004). Stress-induced stacking faults in the Cu/Au interface. J. Phys. Condens. Matter.

[11] Huang Y.-P., Chien Y.-S., Tzeng R.-N., Chen K.-N. (2015). Demonstration and Electrical Performance of Cu–Cu Bonding at 150 °C With Pd Passivation. IEEE Trans. Electron. Devices.

[12] Cha L. (2006). A Metastable HCP Intermetallic Phase in Cu-Al Bilayer Films.

[13] Smet V., Kobayashi M., Wang T., Raj P.M., Tummala R. A new era in manufacturable, low-temperature and ultra-fine pitch Cu interconnections and assembly without solders. Proceedings of the 2014 IEEE 64th Electronic Components and Technology Conference (ECTC).

[14] Sharifi S.H. (2017). Exploration and Optimization of Different SAM (Self-Assembled Monolayer) Deposition Methods to Passivate Copper Microbumps for 3D Stacking. Master’s Thesis.

[15] Tan C.S. (2019). Self-Assembled Monolayer (SAM) Passivation of Copper and Its Application in Wafer Bonding. Encyclopedia of Packaging Materials, Processes, and Mechanics: Set 1: Interconnect and Wafer Bonding Technology. Volume 4: Wafer Bonding Technology.

[16] Lykova M. (2022). Investigation of Cu-Cu Bonding for 2.5D and 3D System Integration Using Self-Assembled Monolayer as Oxidation Inhibitor. Ph.D. Thesis.

[17] Lim D.F., Wei J., Leong K.C., Tan C.S. (2013). Cu passivation for enhanced low temperature (≤300 °C) bonding in 3D integration. Microelectron. Eng..

[18] Nuzzo R.G., Korenic E.M., Dubois L.H. (1990). Studies of the temperature-dependent phase behavior of long chain n-alkyl thiol monolayers on gold. J. Chem. Phys..

[19] Ebbens S., Hutt D., Liu C. (2010). The Thermal Stability of Alkanethiol Self-Assembled Monolayers on Copper for Fluxless Soldering Applications. IEEE Trans. Compon. Packag. Technol..

[20] Hutt D.A., Liu C. (2005). Oxidation protection of copper surfaces using self-assembled monolayers of octadecanethiol. Appl. Surf. Sci..

[21] Love J.C., Estroff L.A., Kriebel J.K., Nuzzo R.G., Whitesides G.M. (2004). Self-Assembled Monolayers of Thiolates on Metals as a Form of Nanotechnology. Chem. Rev..

[22] Tan C.S., Lim D.F., Ang X.F., Wei J., Leong K.C. (2012). Low temperature Cu-Cu thermo-compression bonding with temporary passivation of self-assembled monolayer and its bond strength enhancement. Microelectron. Reliab..

[23] Ghosh T., Dutta A., Lingareddy E., Subrahmanyam C., Singh S.G. Room temperature desorption of Self Assembly Monolayer (SAM) passivated Cu for lowering the process temperature Cu-Cu bonding of 3-D ICs. Proceedings of the Emerging Electronics (ICEE), 2012 International Conference.

[24] Liu C., Hutt D.A. (2006). Fluxless Soldering of Copper Substrates Using Self-Assembled Monolayers for Preservation. IEEE Trans. Compon. Packag. Technol..

[25] Carbonell L., Whelan C.M., Kinsella M., Maex K. (2004). A thermal stability study of alkane and aromatic thiolate self-assembled monolayers on copper surfaces. Superlattices Microstruct..

[26] Kodama C., Hayashi T., Nozoye H. (2001). Decomposition of alkanethiols adsorbed on Au (1 1 1) at low temperature. Appl. Surf. Sci..

[27] Schlenoff J.B., Li M., Ly H. (1995). Stability and Self-Exchange in Alkanethiol Monolayers. J. Am. Chem. Soc..

[28] Keil P., Lützenkirchen-Hecht D., Frahm R. (2007). Investigation of Room Temperature Oxidation of Cu in Air by Yoneda-XAFS. AIP Conf. Proc..

[29] Lykova M., Panchenko I., Künzelmann U., Reif J., Geidel M., Wolf M.J., Lang K.-D. (2018). Characterisation of Cu/Cu bonding using self-assembled monolayer. Solder. Surf. Mt. Technol..

[30] Liu C., Liu A., Su Y., Zhou Z., Liu C. (2021). Nano Ag sintering on Cu substrate assisted by self-assembled monolayers for high-temperature electronics packaging. Microelectron. Reliab..

[31] Ghosh T., Krushnamurthy E., Subrahmanyam C., SivaRamaKrishna V., Dutta A., Singh S.G. Room temperature desorption of Self Assembled Monolayer from Copper surface for low temperature amp; low pressure thermocompression bonding. Proceedings of the 65th Electronic Components and Technology Conference (ECTC).

[32] Lan P. (2012). Wafer-Level Fine Pitch Cu-Cu Bonding for 3-D Stacking of Integrated Circuits.

[33] Biesinger M.C. (2017). Advanced analysis of copper X-ray photoelectron spectra. Surf. Interface Anal..

[34] El-Desawy M. (2007). Characterization and Application of Aromatic Self-Assembled Monolayers. Ph.D. Thesis.

[35] Paul A., Laibinis E., Whitesides G.M. (1992). Self-Assembled Monolayers of n-Alkanethiolates on Copper Are Barrier Films That Protect the Metal against Oxidation by air. J. Am. Chem. Soc..

[36] Moulder J.F., Stickle W.F., Sobol P.E., Bomben K.D., Chastain J. (1993). Handbook of X-ray Photoelectron Spectroscopy.

[37] Deroubaix G., Marcus P. (1992). X-ray photoelectron spectroscopy analysis of copper and zinc oxides and sulphides. Surf. Interface Anal..

[38] Du T., Tamboli D., Desai V., Seal S. (2004). Mechanism of Copper Removal during CMP in Acidic H_2_O_2_ Slurry. J. Electrochem. Soc..

[39] Tajima S., Tsuchiya S., Matsumori M., Nakatsuka S., Ichiki T. (2010). Reduction of Copper Oxide Films by an Atmospheric-Pressure Inductively Coupled Plasma Microjet. Trans. Mater. Res. Soc. Jpn..

[40] Biesinger M.C. (2022). Accessing the robustness of adventitious carbon for charge referencing (correction) purposes in XPS analysis: Insights from a multi-user facility data review. Appl. Surf. Sci..

[41] Arai Y., Nimura M., Tomokage H. Cu-Cu direct bonding technology using ultrasonic vibration for flip-chip interconnection. Proceedings of the 2015 International Conference on Electronic Packaging and iMAPS All Asia Conference (ICEP-IAAC).

[42] Luk C.F., Chan Y.C., Hung K.C. (2002). Development of gold to gold interconnection flip chip bonding for chip on suspension assemblies. Microelectron. Reliab..

[43] Panchenko I. (2013). Process-Dependent Microstructure Changes in Solid-Liquid Interdiffusion Interconnects for 3D Integration.

[44] Lim D.F., Singh S.G., Ang X.F., Wei J., Ng C.M., Tan C.S. Application of Self Assembly Monolayer (SAM) in lowering the process temperature during Cu-Cu diffusion bonding of 3D IC. Proceedings of the IMPACT Conference 2009 International 3D IC Conference.

[45] Lim D.F., Singh S.G., Ang X.F., Wei J., Ng C.M., Tan C.S. Achieving low temperature Cu to Cu diffusion bonding with self assembly monolayer (SAM) passivation. Proceedings of the 2009 IEEE International Conference on 3D System Integration.

[46] Lim D.F., Wei J., Leong K.C., Tan C.S. (2012). Surface Passivation of Cu for Low Temperature 3D Wafer Bonding. ECS Solid State Lett..

[47] Peng L., Li H.Y., Lim D.F., Gao S., Tan C.S. Thermal reliability of fine pitch Cu-Cu bonding with self assembled monolayer (SAM) passivation for Wafer-on-Wafer 3D-Stacking. Proceedings of the 2011 IEEE 61st Electronic Components and Technology Conference (ECTC).

